# Sodium Amytal – No Longer Prescribed, but Still Relevant (And Dangerous!)

**DOI:** 10.1192/bjo.2025.10710

**Published:** 2025-06-20

**Authors:** Conor Brown

**Affiliations:** NIMDTA, Belfast, United Kingdom

## Abstract

**Aims:** Sodium amytal is a barbiturate medication, first synthesised in Germany in the 1920s to treat anxiety and sleep disorders; as well as being used as an anaesthetic. This case report discusses sodium amytal prescription and subsequent dependence in an 82-year-old female with a history of anxiety and agoraphobia. It aims to highlight historical indications, mechanism of action of and potential dangers of cessation. As such, clinical management of withdrawal is discussed including the initial use of a more commonly prescribed barbiturate in place of amobarbital; with the ultimate aim being to cease such dangerous medication and to consider safer alternatives – pharmacological and psychological.

**Methods:** Mrs F was referred to Older Persons Psychiatric Services in 2024. At this time, her GP reported that she was ‘one of only 30 patients in the UK’ to be prescribed sodium amytal, and that it was ‘no longer being produced’. At the time of referral, Mrs F had just a 7-day supply of medication left.

Guidance from the local Medicines Information team had been sought prior to admission. They recommended a phenobarbital taper for seizure prophylaxis when discontinuing other barbiturates, where 100 mg amobarbital was roughly equivalent to 30 mg phenobarbital. Phenobarbital is a long-acting barbiturate which would be more commonly found in practice, given its use in management of epilepsy.

As such, an attempt was made at the safe withdrawal of sodium amytal in the inpatient setting. This was due to the significant risks associated with abrupt withdrawal, including seizure, hallucinations and cardiovascular collapse tending to occur after 16 hours of cessation with such risk remaining up until approximately 5 days.

Mrs F had run out of medication on the day that she was admitted, and given the aforementioned risks, a thorough plan was developed to ensure close monitoring. She was commenced on 1:1 special observations initially due to the risk of seizures. Her clinical observations were performed on a regular basis and a CIWA (Clinical Institute Withdrawal Assessment for Alcohol) score was also performed hourly to monitor withdrawal severity. To this end, lorazepam was prescribed at a dose of 1 mg every 6 hours, as well as an ‘as required’ dose of 1 mg prescribed for when CIWA score was >8.

At consultant review the following day, Mrs F was commenced on phenobarbital at a dose of 30 mg QDS + 30 mg PRN to a maximum of 150 mg/day, continuing on regular lorazepam at 1 mg QDS. Of note, this is slightly lower than the aforementioned recommendation which would equate to 162 mg/day. Subsequently, owing to increasing agitation and anxiety, Mrs F’s dose was increased to 60 mg mane, 30 mg TDS plus 30 mg PRN and her dose of lorazepam was reduced to 1 mg TDS. She was discharged on both medications, 9 days after admission.

**Results:** The treating team discussed the preferred option to follow on from the withdrawal of amobarbital with subsequent tapering of phenobarbital, given that it shares many of the same risks as the former. Mrs F however was not keen to change medications again at present, owing to her age and ongoing anxiety. In fact, at a subsequent outpatient review, Mrs F reported that she felt that she would require more lorazepam to cope with ongoing anxiety, however this was not facilitated. In terms of patient safety, it should be highlighted that the patient was discharged on 2 addictive, sedative medications (even if the lorazepam was a short-term measure), which could prove challenging to rationalise moving forward and perhaps gives an insight into the ongoing difficulties faced by outpatient teams in terms of cessation of addictive medicines. Importantly, the patient would not however experience the same supply issues currently with phenobarbital. It should also be noted that she had declined psychological therapy with the Community Mental Health Team for Older Persons.

**Conclusion:** The aims of this report were to highlight the use and action of the barbiturate class of medications, whilst also discussing difficulties with supply issues, dependence and the associated potential for withdrawal. Ensuring patient safety is paramount, and in many cases patients who are prescribed this class of medications for a prolonged period of time require inpatient management to allow for a safe transition to an alternative medication.

